# *Desmodium molliculum* (Kunth) DC., an Andean medicinal plant: DNA barcoding and HPLC fingerprint for species discrimination and evaluation of its pharmacological potential

**DOI:** 10.3389/fpls.2025.1612556

**Published:** 2025-07-24

**Authors:** Eugenia Peñaherrera, Josue Sarmiento-Pacurucu, Efrén Santos-Ordóñez, Alexandr Kachatryan, Nancy Cuzco, David Vanegas, Jessica Calle-López, Liliana Villao-Uzho, Yvan Vander Heyden, Isabel Wilches, Fabián León-Tamariz

**Affiliations:** ^1^ Department of Biosciences, Group of Medicinal Plants and Natural Products, Faculty of Chemistry, Universidad de Cuenca, Cuenca, Ecuador; ^2^ Faculty of Life Sciences, ESPOL Polytechnic University, Escuela Superior Politécnica del Litoral, ESPOL, Guayaquil, Ecuador; ^3^ Department of Analytical Chemistry, Applied Chemometrics and Molecular Modelling, Vrije Universiteit Brussel (VUB), Brussels, Belgium

**Keywords:** *Desmodium molliculum*, *Desmodium adscendens*, DNA barcoding, HPLC fingerprint, antioxidant, Raci, apigenin flavonoids

## Abstract

In the Ecuadorian traditional medicine, two species of the Desmodium genus, *D. adscendens* and *D. molliculum*, are used interchangeably for the treatment of various ailments, particularly those related to inflammatory processes, wound healing, stomach ulcers and liver disorders. Despite the extensive knowledge and characterization of *D. adscendens*, there is limited information regarding *D. molliculum*. This highlights the necessity for the development of analytical tools that facilitate the differentiation between these two species and the characterization of the latter. The tools were developed and evaluated at two distinct levels: genetically, using the DNA barcoding technique, and analytically, using chromatographic fingerprinting. Additionally, the antioxidant potential of the samples was evaluated through the establishment of the RACI index, based on various *in vitro* evaluation techniques. *De novo* genetic DNA barcodes were obtained for *D. molliculum* and the phylogenetic analysis separated them from those obtained from *D. adscendens*, demonstrating that the *trnH-psbA*, *matK*, and *ITS1* markers are the most effective for differentiating between the species. Additionally, the antioxidant potential of *D. molliculum* was found to be higher than that of *D. adscendens*. The apigenin 8-C-glucoside (vitexin), together with tannic and chlorogenic acids have been pointed by HPLC fingerprinting analysis as responsible for this pharmacological activity.

## 1 Introduction

The Desmodium genus belongs to the Fabaceae family, which is widely distributed worldwide in tropical and subtropical zones. Many members of this genus have a long history of ancient uses, especially medicinal ones. In a recent review, 56 species of this genus were recognized as medicinal, reporting more than 150 different uses or applications (Cueva-Chamba et al., 2023). Species such as *D. styracifolium* (Osbeck) Merr., *D. gyrans* (L.f.) DC and *D. triquetrum* L were incorporated in 2010 into the Pharmacopoeia of the People´s Republic of China (Commission Chinese Pharmacopoeia, 2010). Also, *D. gangeticum* DC was registered in the Ayurveda Pharmacopoeia of India (Government of India et al., 2008). These species have been used to treat various ailments, including inflammation, rheumatism, pyrexia, dysentery, wounds, cough, malaria, hepatitis, hemoptysis, obesity, flu, sore throat, emesis, worm infestation, dysuria, metabolic disorder, asthma, and disorders due to poison (Government of India et al., 2008; Ma et al., 2011). In African traditional medicine, the species *D. velutinum* is reported as useful for many therapeutic purposes, such as antidiarrheal, antipyretic, antiinflammatory, antinephrolithiasis, antibacterial and hypoglycemic in diabetic Wistart rats (Nkwocha et al., 2022).

Because of the important role of this genus in traditional medicine, different studies about their phytochemical composition have been published, reporting more than 200 different molecules, characterized mainly as flavonoids and alkaloids, followed by terpenoids, steroids, phenols, phenylpropanoids, glycosides and volatile oils (Ma et al., 2011). Within this important plant genus, *Desmodium adscendens* has received special attention. This plant is distributed into tropical and subtropical areas around the world and has been used as medicinal plant for the treatment of different ailments and diseases, including muscle cramps, tendinitis, spinal pain, epilepsy, jaundice, hepatitis, bronchitis, hypertension, asthma, allergic reactions and eczema (Seriki et al., 2019; Manzione et al., 2022). Studies about the phytochemistry of *D. adscendens* showed that its main metabolites correspond to soyasaponins, flavonoids and phenolic compounds (caffeic acid, quercetin, p-coumaric acid, epicatechin, and rutin) and simple heterocyclic alkaloids (Muanda et al., 2011). Baiocchi et al. (Baiocchi et al., 2013), reported the structural elucidation of 4 soyasaponins, 4 alkaloid derivatives, and various flavonoids, mainly apigenin and kaempferol glycosides. In addition, Van Dooren et al. (Van Dooren et al., 2018) identified other flavonoid molecules, mainly vicenin-2, isoschaftoside, schaftoside, 2″-O-xylosylvitexin, 2″-O-pentosyl-C-hexosyl apigenin and 2″-O-glucosyl-vitexin in freeze-dried plant decoction.

Studies on the genetics of *D. adscendens* have also been carried out by characterizing it molecularly. DNA molecular barcoding accessions of species obtained from samples from New Caledonia, Tanzania and Mexico for the markers trnH-psbA, rbcL and ITS1, can be found in the NCBI database (Jabbour et al., 2018; Veldman et al., 2020).

**Figure 1:**
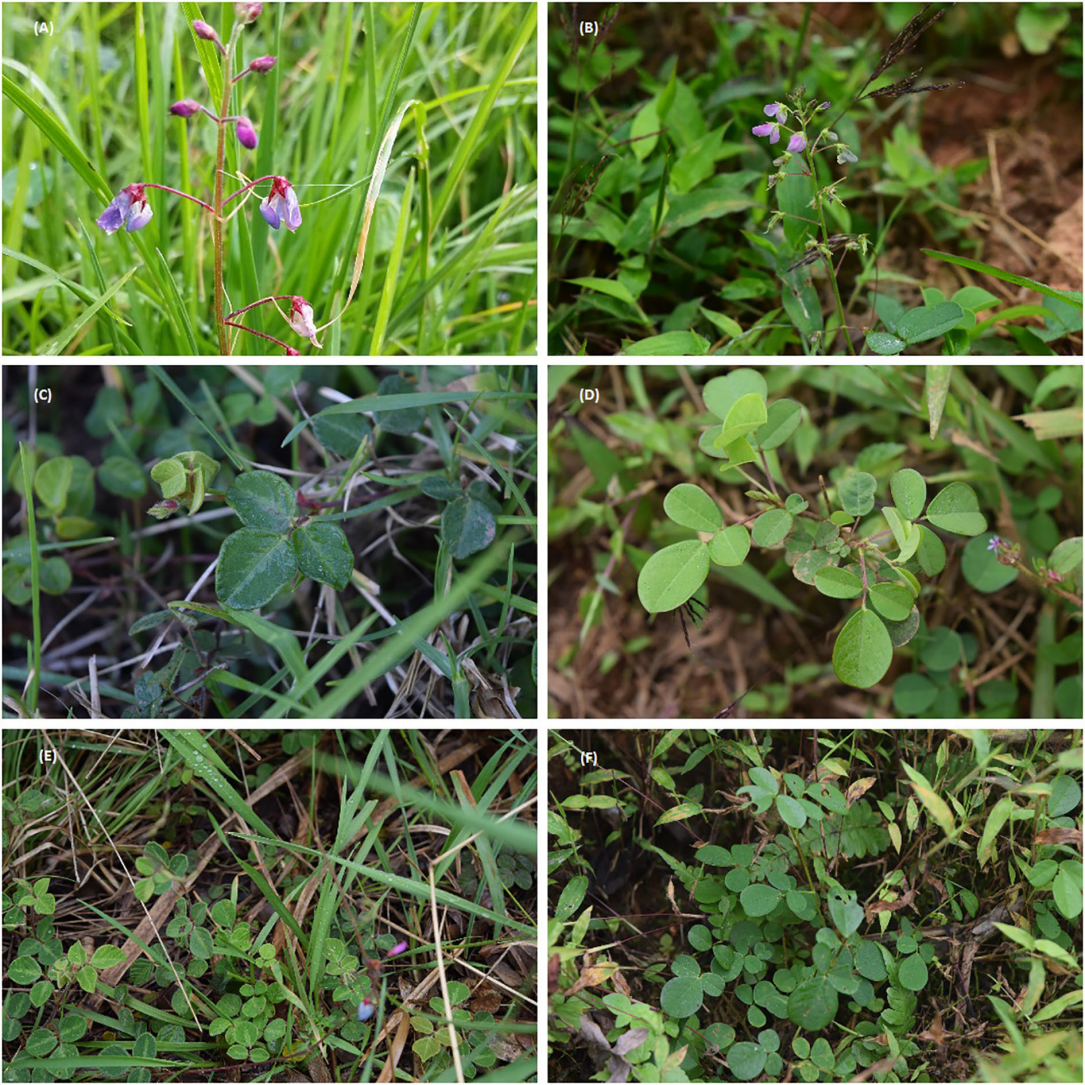
The two Desmodium species *Desmodium adscendens (*DA*)* and *Desmodium molliculum* (D. molliculum), **(A)** flowers on pedicels DM, upper left; **(B)** flowers on pedicels DA, upper right; **(C)** compound leaves with stipules DM, middle left; **(D)** compound leaves with stipules DA, middle right; **(E)** natural habitat for DM, lower left box; and **(F)** habitat for DA, lower right box.

In Ecuador, twenty-one species of the *Desmodium* genus have been described. They were reported at different provinces in the four Ecuadorian regions (including Galapagos), at altitudes between 0 to 3500 meters above sea level (Jorgensen and Leon-Yanez, 1999). *D. adscendens* and *D. molliculum* are the main species recognized as medicinal in Ecuadorian ancestral practices. The first one grows at altitudes between 0-2000 m.a.s.l., while *D. molliculum* does between 1500-3500 m.a.s.l. This factor causes that many people in the Coast, Andes and Ecuadorian Amazon regions confound them and use indistinctly. As can be observed in **Figure 1**, both species share similar morphology, and receive the same common names, such as “hierba del infante”, “hierba del dedo”, “pega-pega” or “hierba de ángel”. Referring to traditional uses, both plants are reported to treat similar conditions, such as inflammatory processes, wound healing, burns, pimples and skin irritations. Internally, their extracts are used to relieve stomach ulcers, liver disorders, and as blood purifier. The main forms of preparation are infusions, decoctions, or poultices (De la Torre et al., 2008, p.; Olascuaga-Castillo et al., 2020; Ríos et al., 2007; Barreto and Bonilla, 2017).

Despite the indistinct traditional use of these two species and their morphological similarities, as mentioned before, the fact that they grow under different environmental conditions and geographic altitudes increases the possibility that they exhibit a qualitatively and quantitatively different phytochemical profile of secondary metabolites that should be inquired since their medicinal benefits could also differ (Van Dooren et al., 2018; Olascuaga-Castillo et al., 2020). Besides, no molecular DNA barcode is available for *D. molliculum*, a factor which, added to the lack of studies related to *D. molliculum*, shows the need to develop analysis methods that become tools to characterize this important medicinal plant species.

The objectives of this research are to develop differentiation criteria for these two species through their chromatographic profiles and genetic differentiation through DNA barcoding of trnH-psbA, rbcL, matK, ITS1 and ITS2 loci. Further, also to establish an initial phytochemical comparison between both species through the correlation between their antioxidant activity and their chromatographic fingerprints.

## 2 Materials and methods

### 2.1 Reagents

Folin-Ciocalteu’s phenol reagent, anhydrous sodium acetate, anhydrous sodium carbonate, gradient grade acetonitrile for liquid chromatography, glacial acetic acid, ethanol, methanol for analysis, and HPLC gradient grade methanol were purchased from Merck (Darmstadt, Germany). Gallic acid, (+/-)-6-hydroxy-2,5.7,8-tetramethyl-chromone-2-carboxylic acid (Trolox), iron III chloride 97% reagent grade, 2,2‐diphenyl‐1‐picrylhydrazyl, 2,2´-azino-bis (3-ethylbenzothiazoline-6-sulfonic acid) diammonium salt, and 2,4,6-Tris(2-pyridyl)-s-triazine were purchased from Sigma-Aldrich (St. Louis, MO, USA). Hydrochloric acid was acquired from Fisher Scientific (New Jersey, USA). Water type I was obtained with a Wasserlab Automatic Plus 1 + 2 water purifier (Barbatáin, Spain) for HPLC analysis.

### 2.2 Methods

#### 2.2.1 Plant collection

Plants specimens of both species were collected according to Good Collection Practices Guidelines (World Health Organization, 2003) in different places of the Azuay and El Oro provinces. Plants were collected under the MAE-DNB-CM-2015–0018 research permission. These two Ecuadorian provinces were selected taking into account different altitudes, in order to have a high variability and to evaluate similarities and differences in plant phytochemical composition and possibly *in vitro* activity. Plant vouchers were prepared and deposited at Herbarium Azuay (HA) for botanical characterization.

#### 2.2.2 Plant extraction

Prior to the preparation of extracts, plant aerial parts (leaves and leaf-stalks) of *D. adscendens* and *D. molliculum* were dried in a PRO-3 drying stove (Cuenca, Ecuador) at 40°C for 24-36 hours until a constant weight was achieved. The dried materials were pulverized prior to extraction. Percolation was selected as extraction method using methanol as solvent (Wilches et al., 2021). The obtained extracts were concentrated in a rotavap Heidolph Laborota 4000 ^®^ (Schwabach, Germany) under a stream of nitrogen. Finally, extracts were freeze-dried using a Labconco Freezone 2.5 (Kansas city, MO, USA). The extracts were stored at -20°C until analysis.

#### 2.2.3 DNA barcoding

##### 2.2.3.1 DNA extraction and polymerase chain reaction

Three individual plants for each Desmodium specie were collected for DNA barcoding analysis. The codes DM1. DM2 and DM3 belongs to the three biological replicates for *D. molliculum*; while the codes DA1, DA2 and DA3 refers to the three biological replicates for *D. adscendens.* Leaves from *D. molliculum* and *D. adscendens* were pulverized with liquid nitrogen and stored at -80°C until DNA extraction. DNA extraction was performed in three biological replicates for each species. The extraction was performed with the CTAB protocol (Pacheco Coello et al., 2017). The quantity and quality of extracted DNA was evaluated by absorbance measurements and gel electrophoresis. For the gel electrophoresis, 5 µL was loaded onto a 1.5% agarose gel to check for amplicons. Once expected amplicons were detected, the remaining 25 µL of the PCR reaction was sent for PCR purification and commercial sequencing to Psomagen (Rockville, Maryland, USA). PCR was performed as described by Bustamante et al. (Bustamante et al., 2019) using 0.5 µM of each primer (**Table 1**) with a reaction volume of 30 µL. GoTaq^®^ master mix was used at a final concentration of 1x according to the manufacturer instructions. PCR conditions were as follows: 95°C for initial denaturation; 35 cycles of 95°C for 30 s, 60°C (for *trnH-psbA* and *rbcL*) or 56°C (for *matK*, ITS1 and ITS2) for 30 s, 72°C for 90 s; and final extension of 72°C for 5 min.

**Table 1:** Primers^1^ for markers *trnH-psbA*, *rbcL*, *matK*, ITS1 and ITS2.

Locus	Primer pairs	Sequence	Reference
*trnH-psbA*	*trnHf*	GTTATGCATGAACGTAATGCTC	(Basak et al., 2019)
	*psbA3*	CGCGCATGGTGGATTCACAATCC	
*rbc*L	*rbcLA*_F	ATGTCACCACAAACAGAGACTAAAGC	(Costion et al., 2011)
	*rbcLA*_R	GTAAAATCAAGTCCACCRCG	
*mat*K	*matK*_3FKIMf	CGTACAGTACTTTTGTGTTTACGAG	
	*matK*_1R_KIMR	ACCCAGTCCATCTGGAAATCTTGGTTC	
ITS1	ITS 5a F	CCTTATCATTTAGAGGAAGGAG	(Chen et al., 2010)
	ITS 4 R	TCCTCCGCTTATTGATATGC	
ITS2	S2F	ATGCGATACTTGGTGTGAAT	
	S3R	GACGCTTCTCCAGACTACAAT	

Adapted from “Morphological and molecular barcode analysis of the medicinal tree *Mimusops coriacea* (A.DC.) Miq. Collected in Ecuador” (Bustamante et al., 2019).

##### 2.2.3.2 Bioinformatic analysis

MEGA11 (RRID: SCR_000667) (Tamura et al., 2021) was used for processing the sequences. Forward and reverse sequences for each amplicon were used to form a consensus sequence. Sequences were used for Blast analysis (Zhang et al., 2000) in the GenBank non-redundant nucleotide database. Sequences with similarities found in the analysis were selected to develop a phylogenetic tree for each DNA barcode. All sequences were trimmed at the ends to contain the same regions and then, aligned by the multiple alignments generating program MUSCLE (RRID: SCR_011812). Promptly, the recommended model was used for each tree. A total of 100 replicates were performed using the bootstrap test.

#### 2.2.4 *In vitro* antioxidant capacity

##### 2.2.4.1 Total polyphenols

The Folin-Ciocalteu assay reported by (Singleton et al., 1999) was used. Briefly, 200 µL of a 10% Folin-Ciocalteu solution prepared with ultra-pure distilled water was placed in test tubes, then 100 µL of the extracts (1 mg/mL) was added to each tube and they were left for 5 min at room temperature. Finally, 800 µL of Na_2_CO_3_ solution (700 mM) was added and the tubes were left to stand for 1 h in the dark. After this incubation time, 200 µL of this mixture was placed in a Costar^®^ 96-well flat-bottom plate (Corning, NY, USA) and the absorbance at 765 nm was measured in a H1 Synergy microplate reader (Bioteck instruments, Winooski, USA). The tests were performed in triplicate.

For the calibration curve, gallic acid was used as standard in concentrations of 0.03125, 0.0625, 0.125, 0.25, 0.5 and 1 mg/mL. Results were expressed as mg gallic acid equivalents per g dry drug weight (*DW*) of the plant (mg GAE/g *DW*).

##### 2.2.4.2 2,2‐diphenyl‐1‐picrylhydrazyl radical scavenging assay

The test was performed according to Brand-Williams et al. (Brand-Williams et al., 1995). A stock solution of DPPH (0.2 mM) in methanol was prepared in a sonic bath. One mL DPPH solution was transferred to a test tube, 50 µL extract (1 mg/mL) or methanol (control) and 200 µL methanol were added. The mixture was incubated for 30 min in the dark at room temperature. Finally, 100 µL of each tube was transferred to a 96-well flat-bottom plate and absorbance was measured at 517 nm in a H1 Synergy microplate reader. All samples were tested in triplicate. Trolox was used for the calibration curve in concentrations of 0.44, 0.22, 0.11, 0.055, 0.0275, 0.01375 and 0.006875 mg/mL. Values were expressed as mg Trolox equivalents per g dry drug weight (mg TE/g *DW*).

##### 2.2.4.3 2,2′‐azinobis (3‐ethylbenzothiazoline‐6‐sulfonic acid)/Trolox equivalent antioxidant capacity assay

The test was performed according to Re et al. (Re et al., 1999). The ABTS•^+^ radical solution was prepared with 10 mL of 7 mM ABTS and 10 mL potassium persulfate (2.45 mM) in a 1:1 ratio. The mixture was left in the dark at room temperature for 12-16 h to complete the reaction. Absorbance of the ABTS•^+^ solution was adjusted to 0.7 (± 0.02) at 734 nm with ethanol before use. Then, 40 µL extract (1 mg/mL) or ethanol (blank) was added into the test tubes and then 800 µL ABTS•^+^ solution. The mixture was allowed to stand for 30 min in the dark and finally read in a microplate reader at 734 nm. The assay was performed in triplicate. A Trolox calibration curve was constructed as described before. Values were expressed in mg TE/g *DW*.

##### 2.2.4.4 Ferric reducing antioxidant power

The necessary reagents; acetate buffer (300 mmol/l), 2,4,6-tri(2-piridil)-1,3,5-triazine (TPTZ) solution (10mM), ferric chloride (20 mmol/l) and working FRAP reagent were prepared according to Benzie and Strain (Benzie and Strain, 1996). To perform the assay, to 600 µL working solution, 20 µL extract (1 mg/mL) was added, mixed and read after 30 min in a microplate reader at 593 nm. All samples were tested in triplicate. A Trolox calibration curve was constructed as described before. Values were expressed in mg TE/g *DW*.

##### 2.2.4.5 Relative antioxidant capacity index

The RACI of each plant extract was calculated as the mean of standard scores transformed from the raw data generated with different chemical methods as proposed by (Sun and Tanumihardjo, 2007). For this study, an alternative index, based on the scores of the first principal component derived from Principal Component Analysis (PCA), was also proposed. With this methodological variation, it was expected to obtain an alternative and representative indicator that reflects the comprehensive antioxidant capacity of the analyzed samples.

#### 2.2.5 Development of HPLC fingerprints

##### 2.2.5.1 Equipment

An Agilent 1200 HPLC quaternary pump system (Palo Alto, CA, USA) equipped with an automatic sampler, thermostat and a DAD detector was used. A Merck LiChrocart C18 column (250 x 4 mm; i.d.,5 µm) with a LiChrospher^®^ 100 RP-18 (5 µm) guard column from Merck (Darmstadt, Germany) was used.

##### 2.2.5.2 Sample preparation for HPLC analysis

The stock solutions of all samples for HPLC analysis were prepared at a concentration of 20 mg/mL using methanol HPLC grade as solvent. Working solution of 1 mg/mL was prepared, followed by filtration through a polypropylene membrane 0.45 µm pore size from Titan, (Rockwood, TN, USA). Phytochemical standards were dissolved in pure methanol for a final concentration of 0.1 mg/mL.

##### 2.2.5.3 Chromatographic conditions

To develop the fingerprints of *D. adscendens* and *D. molliculum*, some chromatographic conditions were chosen based on previous studies on *D. adscendens* (Baiocchi et al., 2013; Lee et al., 2020) and an in house experimental work. Briefly, HPLC fingerprints were determined at a temperature of 30°C using a C18 column with a precolumn. The injection volume was 20 µL. The column was conditioned for 30 min at the desired temperature and flow conditions. Wavelengths considered were 254, 280 and 330 nm. The flow rate was 0.75 mL/min. Solvent A contained 0.1% acetic acid in ultrapure water, and solvent B was acetonitrile with 0.1% acetic acid. The gradient (ratio A/B) was: 0-5 min 100/0, 55 min 50/50, 90 min 0/100 and 95 min 100/0, with 15 min for re-equilibration. Total run time was 110 minutes.

Quercetin, quercetin-3-O-β-D-glucuronide, apigenin, apigenin-7-glucoside (apigetrin), apigenin 8-C-glucoside (vitexin), luteolin 4′-methyl ether (diosmetin), luteolin-7-glucoside (cynaroside), luteolin, kaempferol, kaempferol-3-glucoside (astragalin), 4′,7-dihydroxy­iso­flavone (daidzein), isorhamnetin, 5,7-dihydroxyflavone (chrysin), 3,4,5-trihydrobenzoic acid (gallic acid), tannic acid (gallotannin), trans-ferulic acid, caffeic acid, 3-(3,4-dihydroxycinnamoyl)quinic acid (chlorogenic acid) and 5-O-(trans-3,4-dihydroxycinnamoyl)-D-quinic acid (neochlorogenic acid) were purchased from Fluka, Sigma-Aldrich (St. Louis, MO, USA). Phenolic standards were accurately weighted and diluted in pure methanol for a final concentration of 1 mg/mL (stock solutions), dilutions of 1/20 were prepared for each standard. Besides, a total standards solution containing 0.050 mg of each product in 1 mL pure methanol was prepared. Total standards solution and pure solutions were injected under the same chromatographic conditions as plant extracts.

##### 2.2.5.4 Data preprocessing

In the context of chromatographic fingerprinting analysis, it is essential to develop a preprocessed database focusing on three basic steps, noise removal, alignment of peaks in the chromatograms and baseline correction (Wehrens, 2020). Furthermore, in liquid chromatography, it is advisable to subtract the blanks from chromatograms to mitigate any noise caused by the wear of the instrument or the stationary phase (Komsta et al., 2018). Chromatographic correlation was evaluated using Pearson correlation distance (PCD). These PCD´s were analyzed in order to evaluate the need, or not, to align the chromatographic peaks. For the baseline correction, the “fillPeaks” method from the “baseline” package in R was applied in order to improve data quality and facilitate data interpretation, which can become difficult due to changes in mobile phase composition or stationary phase bleed and signal variation due to electronic noise.

##### 2.2.5.5 Similarity analysis

Similarity between chromatograms was assessed by generating a distance matrix (DisM) using Pearson’s correlation coefficients. With this matrix, a hierarchical clustering analysis (HCA) was performed using Ward’s method as clustering criterion. This approach provides a clear view of the relationships between chromatograms and how they can be grouped, based on their similarity (Palacio et al., 2020).

To validate the quality of this hierarchical grouping, two measures were applied: the Cophenetic Correlation Coefficient (CCC) and the Hopkins statistic. The CCC was calculated by creating a cophenetic matrix of the dendrogram and then comparing it with the original similarity matrix through the Pearson’s correlation coefficient. A significantly high correlation between these matrixes would indicate that the original structure has been adequately preserved in the dendrogram. On the other hand, the Hopkins statistic was used to determine the suitability of the data for clustering, looking for values close to zero that suggest a non-uniform data structure and, therefore, suitable for the clustering process (Goodarzi et al., 2013).

##### 2.2.5.6 Building, evaluation and validation of predictive models

In order to correlate and predict the antioxidant capacity (RACI) of plant metabolites present in *Desmodium* extracts, two models using the regression techniques Partial Least Squares (PLS) and Orthogonal Projection to Latent Structures (OPLS) were proposed. For the evaluation of the PLS and OPLS models, the following performance indicators were used: Explained Variability, Coefficient of Determination (R²), Root Mean Square Error (RMSE), Ratio of Performance to Deviation (RPD). The “leave-one-out” technique was used as a cross-validation strategy in order to determine the effectiveness of the regression model, allowing a comprehensive evaluation of its predictive capacity.

##### 2.2.5.7 Correlation between biological activity and chromatographic fingerprint by PLS and OPLS

For the purpose of identifying the possible active metabolites that are responsible for the antioxidant effect, models were built using PLS and OPLS. The PLS is particularly useful when trying to model complex relationships between a set of independent variables (predictors), which, in the present case would be the absorbance values at the specific time interval of each chromatogram, and one or a set of dependent variables (responses), which here is the RACI value (Wehrens, 2020).

The OPLS represents an evolution of the Partial Least Squares regression, and has established itself as a fundamental tool in chemometrics for the analysis of complex data. The key difference between OPLS and PLS is in the treatment of the variability of the predictor data (Wehrens, 2020). While PLS focuses on maximizing the covariance between the independent and dependent variables, OPLS decomposes the variation of predictors into two distinct components: one that is predictive, directly correlated with the response variable, and another that is orthogonal, i.e. not related to the response variable (Ben Ahmed et al., 2016).

## 3 Results and discussion

### 3.1 Plant collection and extraction

**Figure 2:**
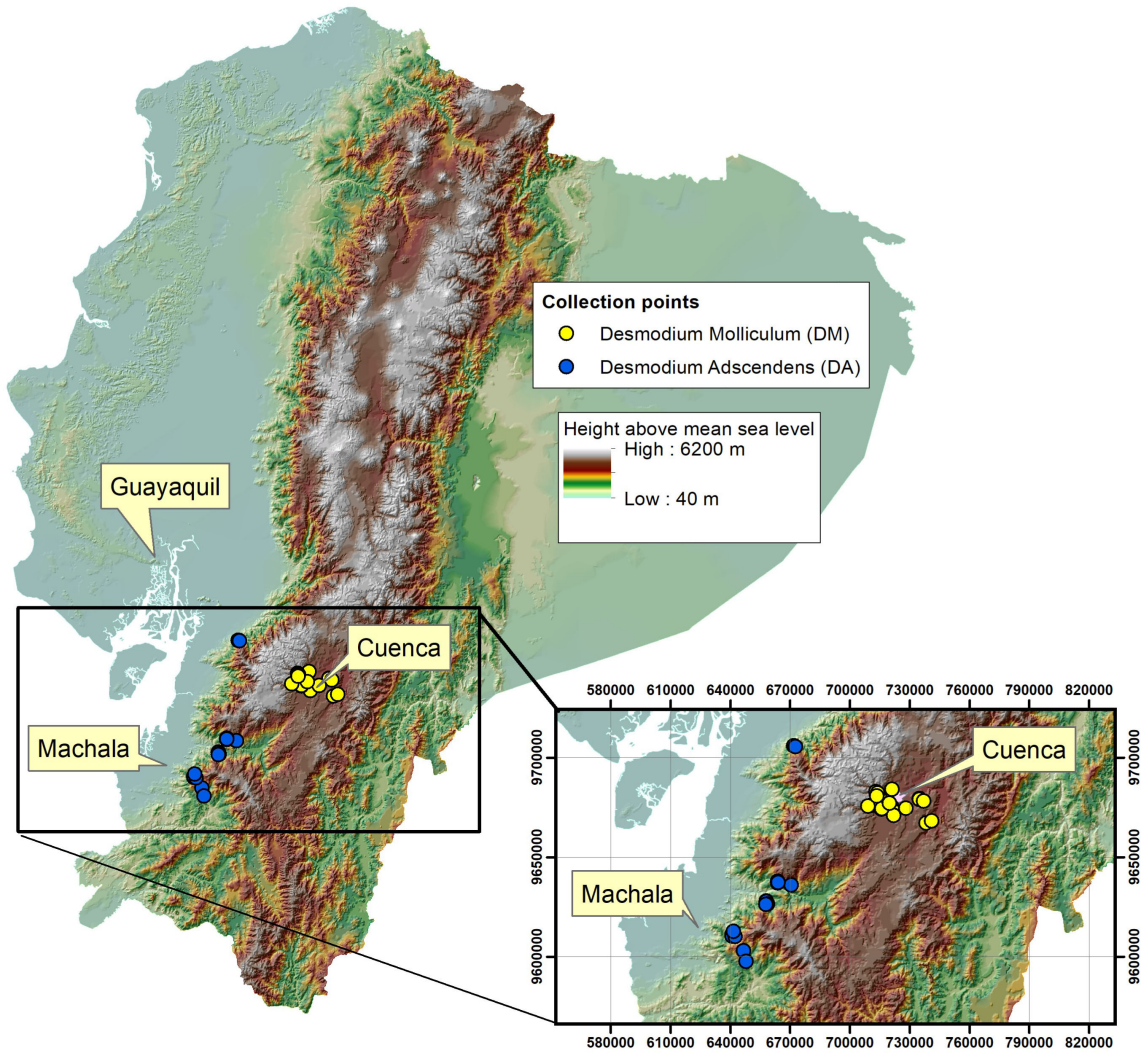
Collection places for *Desmodium adscendens* (DA) and *Desmodium molliculum* (DM).

Twenty samples of *D. adscendens* were collected at altitudes between 1003 to 1482 m.a.s.l, and twenty samples of *D. molliculum* at altitudes between 2553 to 2978 m.a.s.l. Voucher specimens were characterized and deposited in the Herbarium Azuay (HA). **Figure 2** shows the different collection places.

After freeze drying of methanol extracts, the Drug Extract Ratio (DER_NATIVE_) was calculated. For DA, an average DER_NATIVE_ of 8.2:1 was obtained, while for DM, a somewhat higher yield was observed with a DER_NATIVE_ average value of 7.6:1.

### 3.2 DNA barcoding

**Figure 3:**
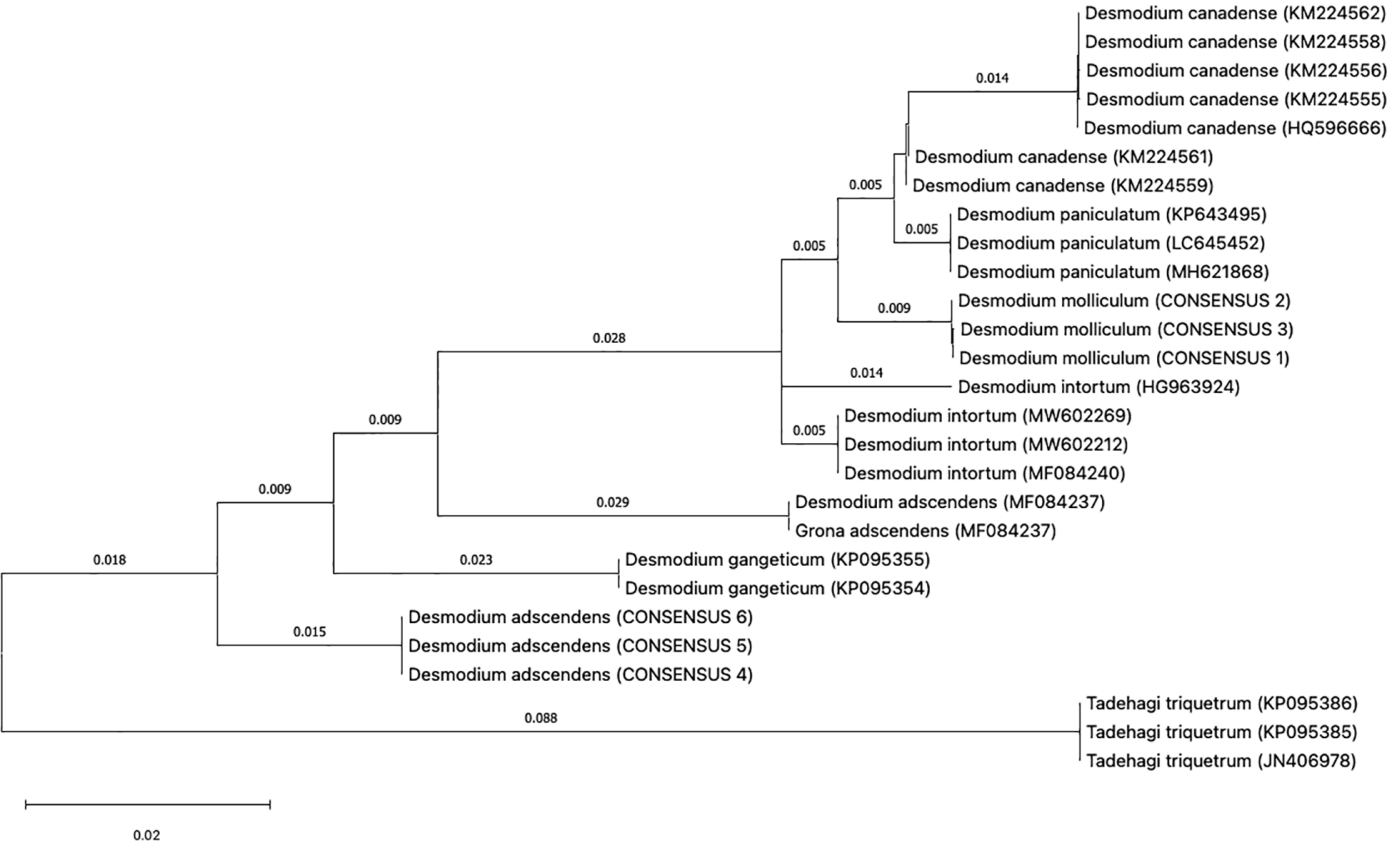
Topology of the *trnH-psbA* original tree.

**Figure 4:**
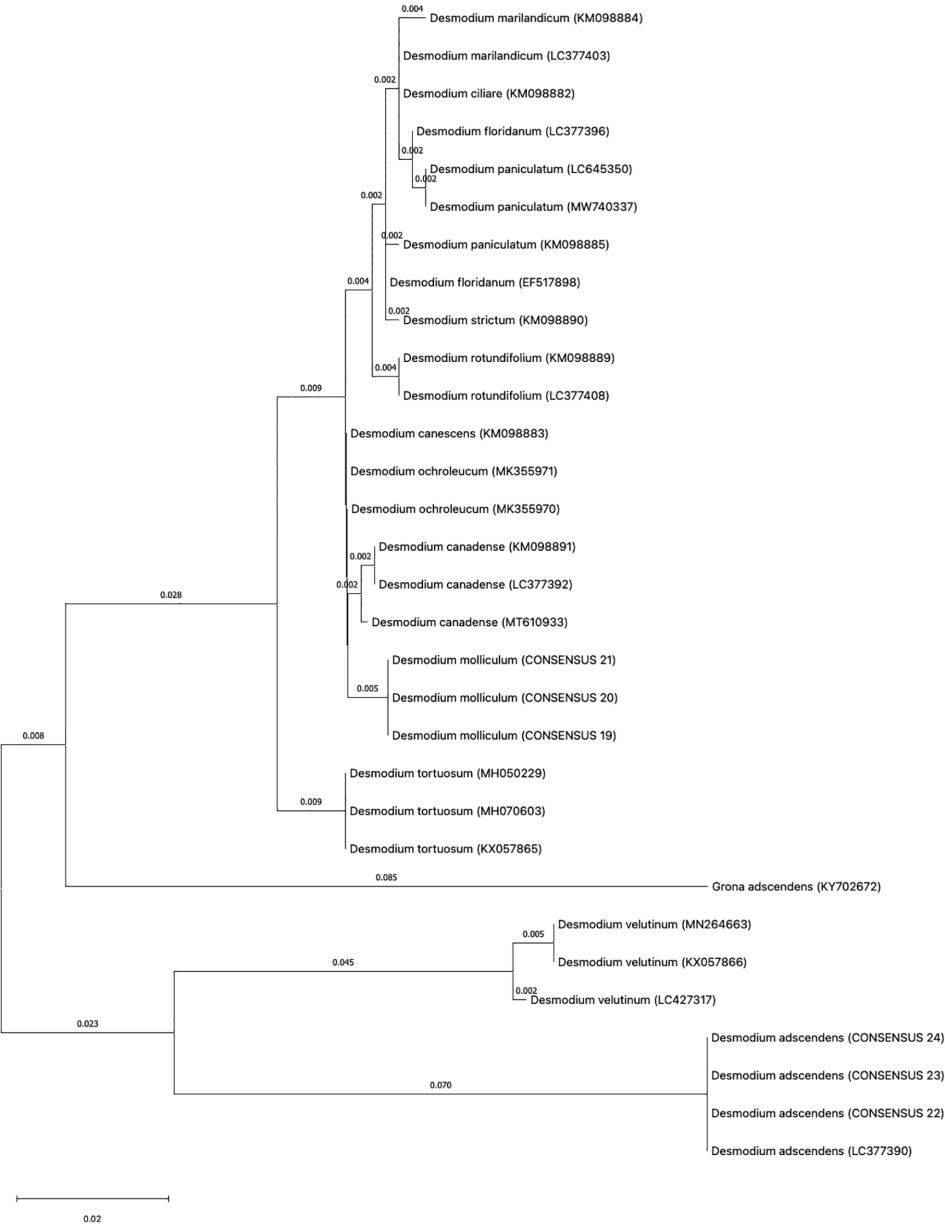
Topology of the ITS1 original tree.

As indicated in materials and methods, three individual plants for each Desmodium specie was analyzed for DNA barcoding. In most DNA barcodes, the similarity with GenBank accessions was with species of the genus Desmodium. Blast analysis, DNA barcode markers, sequences and replicas obtained from *D*. *molliculum* and *D. adscendens* for all markers can be observed in **Table 1**. DNA barcode and phylogenetic analysis of all markers used showed that *D. molliculum* and *D. adscendens* sequences arranged in distinct and separate clades in their original trees (**Figures 3**, **4**; **Supplementary Figures**). It is important to note that the accessions obtained for the DNA barcodes *trnH-psbA*, *rbcL*, *matK*, ITS1 and ITS2 for *D. molliculum* are *de novo* and were elucidated for the first time. Consequently, no accessions for any *D. molliculum* DNA bardcode were found in the NCBI for comparison with the accessions obtained in this investigation.

The distribution of the *D. molliculum* and *D. adscendens* sequences in the trees of all markers, clearly indicates that the collected species are different. *D. adscendes* sequences obtained in this study were compared with other accessions available in the NCBI for the same species. Included accessions were: MF084237 (*trnH-psbA*), KY702612 (*rbcL*) and KY702672 (ITS1) from New Caledonia (Jabbour et al., 2018), MN166747 (rbcL) from Tanzania (Veldman et al., 2020) and LC377390 (ITS1) from Mexico (Ohashi et al., 2018). The *trnH-psbA* tree (**Figure 3**) shows the *D. adscendens* sequences separated from the MF084237 accession, while the *rbcL* topology (**Supplementary Figure S1**) shows all *D. adscendens* sequences sharing the same node. The *D. adcendens* sequences of this study are closer to the Tanzanian (MN166747) accession than the New Caledonian (KY702612) which is in the consequent closest branch. Similarly, the ITS1 tree (**Figure 4**) showed *D. adcendens* sequences in the same clade as the accession LC377390 (Veldman et al., 2020) but, separated from KY702672 (Jabbour et al., 2018). Finally, the ITS2 tree (**Supplementary Figure S2**) separates *D. adscendens* consensus sequences (CS) from all the others samples.

Regarding the *matK* tree (**Supplementary Figure S3**), it is important to mention that the sequences obtained for this locus were not of optimal quality due to drawbacks in the recovery and amplification process. Therefore, the first *D. adscendens* sequence (DA1) had to be removed from the analysis. This locus has been reported as problematic by other authors too, due to its low amplification and recovery success (Nei and Kumar, 2000; CBOL Plant Working Group et al., 2009). This phenomenon is roughly reflected in the topology of the tree, where different branch distances can be observed for the two *D. adscendens* sequences, obtained from the same sample, to the node where they originate.

In addition, bootstrap support in all of the phylogenetic trees was analyzed. The bootstrap value serves as an aid to evaluate the branching pattern of the tree. If it exceeds 95%, it indicates that clades are correctly formed and that their topology is correct (Nei and Kumar, 2000). However, it has been suggested that values over 70% also represents true clades and correct clade formation (Hillis and Bull, 1993).

With these considerations, we found that the markers *trnH-psbA*, *matK* and ITS1 had the highest support percentage being the most apt for Desmodium species discrimination. All three markers had a bootstrap support higher than 95% in all the clades formed for *D. adscendens* CS, while clades formed for *D. molliculum* CS nodes had a support of 91% (*trnH-psbA*), 79% (*matK*) and 93% (ITS1). These findings agree with the literature. According to Hollingsworth et al. (CBOL Plant Working Group et al., 2009), the *trnH-psbA* and *matK* loci have a good discrimination power and offer higher resolution when discriminating angiosperms at the species level. Moreover, Costion et al. (Costion et al., 2011) describes *trnH-psbA* as the most efficient marker to use in species discrimination in poorly known floras. ITS1 is also shown to be useful for species discrimination within the Sapotoideae family in Ecuadorian specimens (Bustamante et al., 2019). On the other hand, the bootstrap support for the rbcL tree, was low for both *D. molliculum* CS (59%) and *D. adscendens* CS (61%) clades, making this marker the least suitable for discrimination between the presented species.

Concerning the ITS2 tree, the bootstrap analysis showed mixed results. The clade for *D. adscendens* CS had a 99% support, while the *D. molliculum* CS clade was supported by only 61%, one of the lowest percentages obtained in this research. Despite the fact that the ITS2 marker has been reported to be successful in identifying specimens to the species level by up to 92.7% (Chen et al., 2010) and other studies on medicinal plants highly recommend the use of the ITS markers for species identification and differentiation (Techen et al., 2014; Zhang et al., 2016; Bustamante et al., 2019; Sarmiento-Tomalá et al., 2020; Soledispa et al., 2021; Vielma-Puente et al., 2025), it was found that is not the best marker for *D. molliculum* identification.

Overall, *D. molliculum* clades were not highly supported by bootstrap analysis, indicating that the topology of its branches may not be correct. These misassignments at species level may occur because of a high genetic similarity between the specimens studied which supports the idea that *D. adscendens* is the farthest relative species from the other Desmodium samples. Nevertheless, these results may also occur due to morphological misidentifications in species with similar phenotype (Loera-Sánchez et al., 2020).

### 3.3 Evaluation of the *in vitro* antioxidant capacity

The results corresponding to the *in vitro* antioxidant evaluation of plant extracts (total polyphenols, DPPH, ABTS and FRAP) are presented in **Supplementary Table S1**. Results corresponding to *D. molliculum* clearly show a higher antioxidant effect than *D. adscendens* in every applied analytical method (*p <* 0.001). However, when it comes to antioxidant capacity, authors such as Sun and Tanumihardjo (Sun and Tanumihardjo, 2007) and Lee et al. (Lee et al., 2020) have suggested that instead of analyzing antioxidant capacity separately by evaluating individual assays (such as those used in this study), and considering that different analytical methods differ in their principles and methodologies to evaluate this complex activity, one might consider the use of a Relative Antioxidant Capacity Index (RACI). This approach allows to integrate the results of various methods into a single numerical value, allowing simple comparison of the antioxidant capacity of a large number of samples (Sun and Tanumihardjo, 2007; Lee et al., 2020).

**Figure 5:**
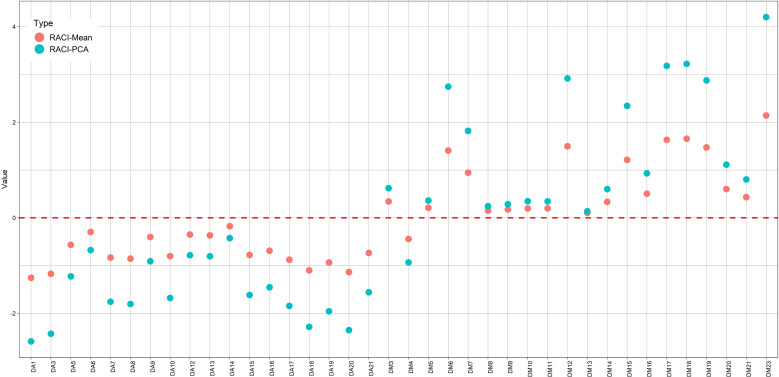
RACI values, calculated as the average of the normalized values obtained from the different methods (•) and based on the scores of the first principal component (PC1) (•).

The RACI is calculated as the average of the standardized scores obtained from the different methods. However, for this study, the analysis of an alternative index, based on the scores of the first principal component derived from Principal Component Analysis (PCA), was also proposed. Basically, this index captures the same information as the previous one, given that it employs a weighting, i.e., a linear combination of the different methods (it has a linear relationship with the RACI, R^2^ = 0.999), to quantify the antioxidant capacity of the samples. With this methodology, an increase in the visualization of the separation between the different levels of antioxidant capacity was generated; in addition, it provides information on the loadings of each method for the new index as opposed to the one generated (RACI) by averaging. The result of the analysis of the loadings (DPPH 0.47; ABTS 0.49; FRAP 0.53 and Total polyphenols 0.51) showed a very small increase-irrelevant for FRAP. However, its effect on the visualization of the separation of antioxidant activity index levels is remarkable, as can be seen in **Figure 5** and **Supplementary Figure S4**.

### 3.4 HPLC fingerprinting analysis

#### 3.4.1 Data preprocessing

Each plant extract was analyzed in duplicate, leading to the generation of 40 chromatograms for each of the two, presumably different, plant species. After blank correction of the fingerprints, to evaluate the reproducibility of the results, an analysis using Pearson’s correlation coefficient was carried out between the replicates. The obtained score (*r* > 0.93) discarded the need to develop any treatment for peak alignment and allowed the consolidation of data into a mean chromatogram.

**Figure 6:**
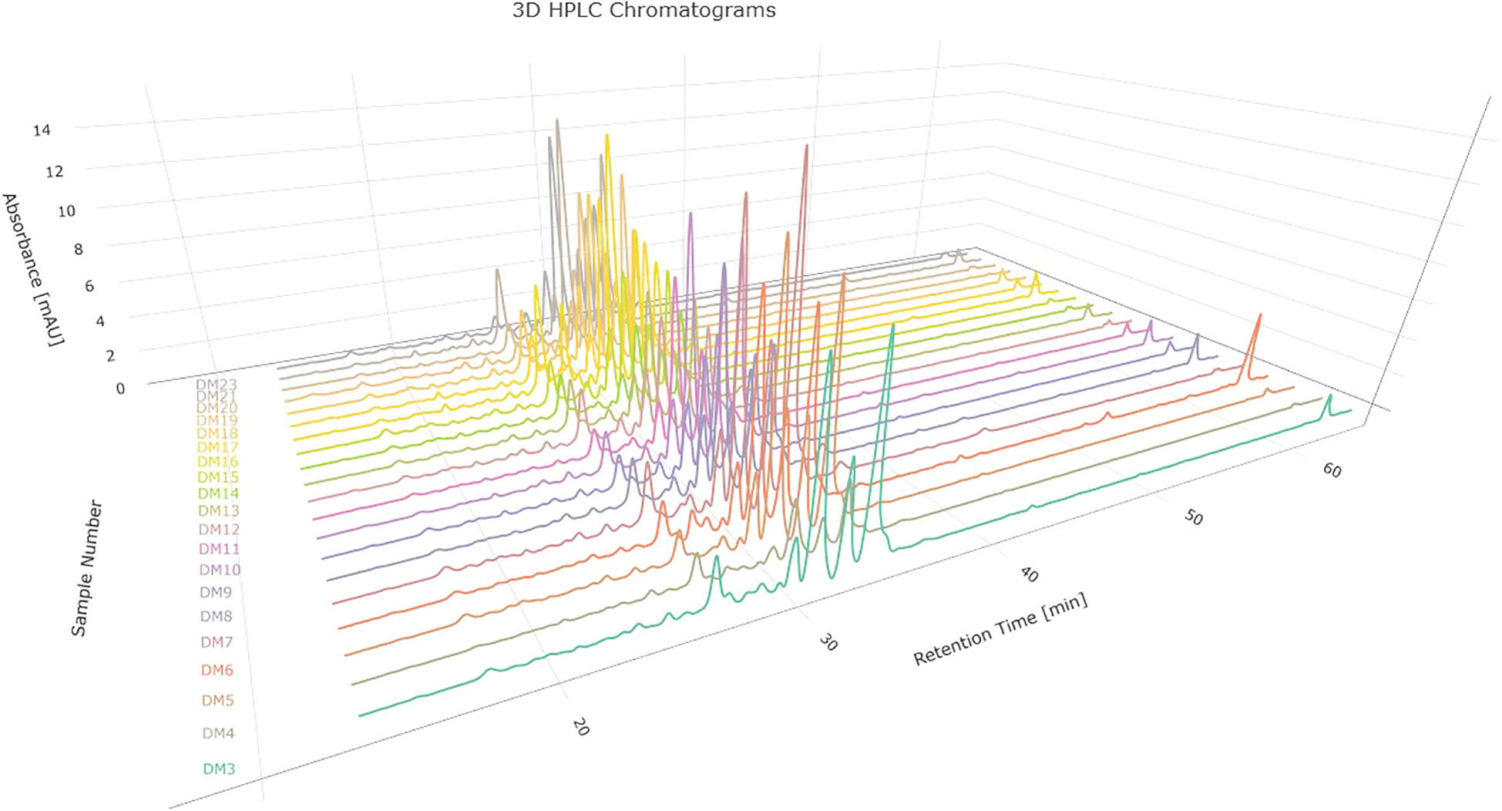
Chromatograms corresponding to *D. molliculum* (D. molliculum) measured at 330nm.

On the other hand, by visually evaluating the chromatograms corresponding to the specimens and blanks, the time interval for the subsequent fingerprint analysis was determined. This interval covered a specific period of time between 13.2 and 64.5 min, which represents a total of 7696 absorbance measurements taken in that time interval. Chromatograms corresponding to *D. molliculum* at 330nm are showed in **Figure 6**.

**Figure 7:**
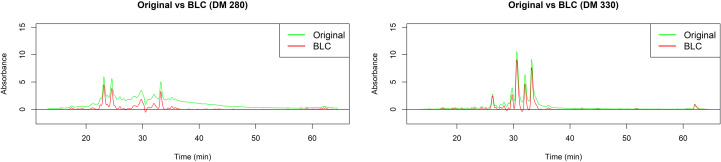
Baseline correction (BLC) for *D. molliculum* (DM) chromatograms measured at 280 and 330nm.

Once the reproducibility between replicates of each extract was confirmed, the median chromatogram for each specie was obtained. A baseline correction was applied to these median chromatograms. This procedure is important in order to separate the analyte signal of interest from signal which arises due to changes in mobile phase composition, stationary phase bleed, or electronic noise (Wehrens, 2020). Performing this correction ensures that the peaks and valleys in the chromatograms accurately reflect the substances present in the analyzed samples, which in turn increases the reliability of interpretations and conclusions. Results for *D. molliculum* extracts are presented in **Figure 7**.

#### 3.4.2 Similarity analysis

Similarity assessment of complex chromatographic profiles, such as those from medicinal plants or herbal products, is important as a potential tool for their identification. An unsupervised data analysis technique was used. To the obtained matrix, a hierarchical clustering analysis (HCA) was performed using Ward’s method as clustering criterion. The strength of this method lies in the minimization of the variance within each group, analyzing all possible combinations and selecting the one with the smallest least squares error (Aldas Manzano and Jimenez, 2017).

**Figure 8:**
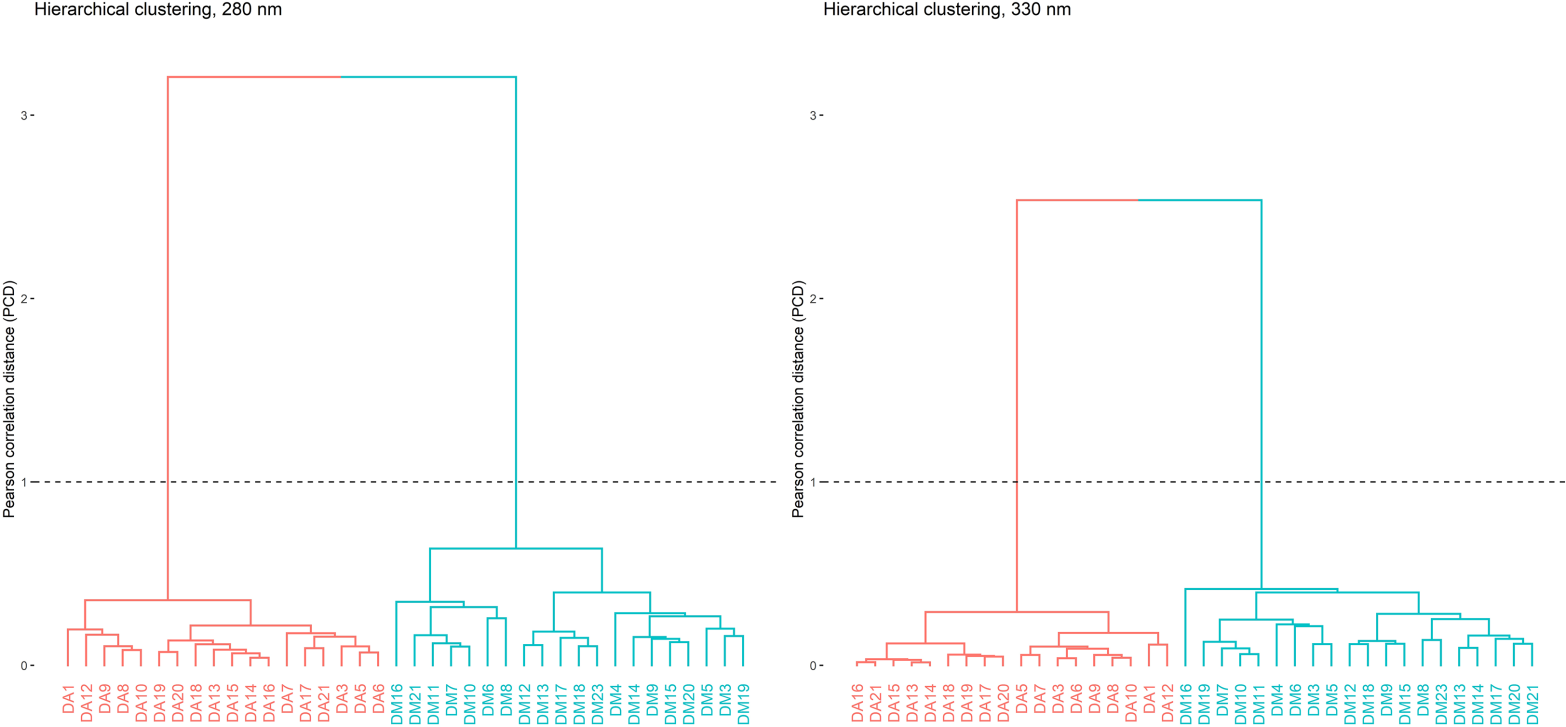
Clusters corresponding to *D. adscendens* (DA) and *D. molliculum* (DM) measured at 280 and 330nm.

Two clusters, corresponding to *D. molliculum* and *D. adcendens* were identified. In order to improve the visual representativeness of the dendrograms, increasing their interpretation and highlighting the relationships between samples, a transformation in the similarity matrix, applying square root extraction in order to reduce the distances between the chromatograms, was applied (Chang et al., 2001). As can be seen in **Figure 8**, a clear differentiation between both plant species was observed. The two wavelengths used for the data analysis showed excellent values confirming the results, both for the Hopkins coefficient (0.17 for 280 nm, 0.15 for 330 nm) and for the Cophenetic correlation (0.974 for 280 nm, 0.968 for 330 nm).

#### 3.4.3 Building, evaluation and validation of predictive models

For the independent variables (predictors), the variability explained by the PLS model at 280 nm during calibration was 94.9% (with two latent variables, PC1: 85.1%, PC2: 9.8%) of the total variance. The variability explained by the model for the dependent variable (response) during calibration, was 76.7% of the variance in the dependent variable (with the same latent variables). Those results, together with those from cross validation, ​​indicate a good predictive capacity of the model.

The Mean Squared Error for calibration is 0.884 and for cross validation 0.980. These values ​​quantify the average of the squared differences between the observed values ​​and the values ​​predicted by the model. Lower values ​​indicate better fits of the model to the data. The Performance to Deviation Ratio is 2.10 for calibration and 1.89 for cross-validation. An RPD higher than 2.0 is considered good for calibration, indicating that the model has a robust predictive capacity (Chang et al., 2001). In cross-validation, the RPD is slightly lower than 2.0, suggesting that the predictive ability of the model is good, but it might be improved.

At 330 nm, the PLS model, configured also with two latent variables, demonstrated strong explanatory power by describing 97.4% of the variability in the independent variables and 60.7% in the dependent variable within the calibration sample. These results point to a notable ability to capture the underlying dynamics between predictor variables and the response. In contrast, the OPLS model, although with one latent variable, showed a slightly reduced explanation of the independent (96.1%) and dependent (58.9%) variability. The OPLS exhibited a similar result in cross-validation with an R² of 0.539 compared to 0.527 for PLS.

In the evaluation of predictive accuracy, the RMSE of the PLS model (1.148 in calibration and 1.258 in cross-validation) was marginally worse than that of the OPLS (1.174 in calibration and 1.242 in cross-validation), implying a slight advantage in terms of precision for the OPLS predictions. The differences are minimal, suggesting that both models are comparable in terms of accuracy and are competent for the analysis used in the present research.

#### 3.4.4 Assignment of active compounds by PLS and OPLS

**Figure 9:**
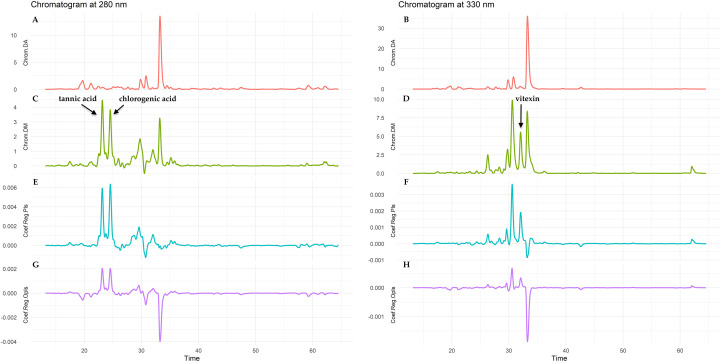
Predictive models based on antioxidant capacity (RACI) applying Partial Least Squares (PLS) and Orthogonal Projection to Latent Structures (OPLS) techniques for *D. adscendens* (DA) and *D. molliculum* (DM) extracts measured at 280 and 330 nm. **(A, B)** chromatograms of DA at 280 and 330 nm; **(C, D)** chromatograms of DM at 280 and 330 nm; **(E, F)** regression coefficients of PLS models; **(G, H)** regression coefficients of OPLS models.

The main focus of these models was to indicate those peaks in the fingerprints that are potentially responsible for the antioxidant activity. This prioritization was based on the observation that *D.molliculum* extracts exhibit a significantly higher antioxidant capacity compared to *D. adscendens* ones. In order to achieve the proposed objective, a detailed analysis of the regression coefficients was carried out. The central focus of this study was to identify those coefficients that contribute significantly (from an analytical point of view) to the increase in the response variable. As can be observed in **Figure 9**, a specific interval stands out between 20 and 35 min of the chromatograms at 280 and 330 nm, which arouses particular interest due to the presence of differentially expressed metabolites between the *D. adscendens* and *D. molliculum* samples.

On the regression plots, the coefficients indicating peaks corresponding to potential antioxidant compounds or to those representing a similar behavior, i.e. that are present at high concentration when the antioxidant activity is high, are positive as the RACI results increase with increasing activity. Positive regression coefficients represent compounds that show a similar behavior to the antioxidant activity.

Performing a more detailed analysis of the aforementioned time interval, and considering the different phytochemical standards used in the present research, it is noted that there are two metabolites identified as active in the *D. molliculum* at 280nm, which correspond to tannic acid and 3-(3,4-dihydroxycinnamoyl) quinic acid (chlorogenic acid), and differ substantially from those eluting in the *D. adscendens* corresponding extracts, as can be seen in **Figure 9**. Furthermore, the PLS and OPLS models mark the same peaks as being responsible for antioxidant activity. At 330nm, both models pointed to the same chromatographic peaks as responsible for the antioxidant activity. At this wavelength, the injected phytochemical standards allowed determining the presence of apigenin 8-C-glucoside (vitexin) in higher concentration in *D. molliculum* than in *D. adscendens*, but not the identity of the preceding chromatographic peak, present in both species and for which both models assigned an important relationship with the observed antioxidant effect.

After literature review about the phytochemistry of the *Desmodium* genus, polyphenols, more specifically flavonoids, are pointed as responsible for many of biological activities attributed to different species (Tsai, 2011). Flavonoids are considered as an indispensable component in a variety of nutraceutical, pharmaceutical, medicinal and cosmetic applications. This is attributed to their antioxidant, antiinflammatory, or anticarcinogenic properties coupled with their capacity to modulate key cellular enzyme function (Panche et al., 2016; Wen et al., 2020). The presence of derivatives of the flavone apigenin and flavonol kaempferol were reported as the more significant polyphenolic compounds in *D*. *adcendens*, while quercetin derivative was also identified (Baiocchi et al., 2013). However, based on the applied fingerprints and predictive models, only vitexin, and phenolic acids as tannic and chlorogenic showed an important contribution to the observed antioxidant effect. Analysis with complementary techniques as mass spectrometry are needed for a more complete compositional elucidation (Baiocchi et al., 2013).

## 4 Conclusions

Despite the physical similarity of the two species analyzed in the present study, the series of genetic and chromatographic tools employed allowed generating valid criteria for a correct differentiation and classification of *D. molliculum* and *D. adscendens*. When considering genetic barcoding analysis, the markers *trnH-psbA*, *matK* and ITS1 showed the highest efficiency for discriminating between Desmodium species, while *rbcL* showed the poorest results.

The chromatographic-fingerprint analysis allowed an adequate differentiation between the two species through HCA. In addition, the use of RACI index, based on the scores of the first principal component (PC1) allowed greater efficiency in the visualization of antioxidant capacity of plant extracts.

For the identification of the metabolite(s) potentially responsible for Desmodium antioxidant activity, the present study was based on the analysis of regression coefficients derived from partial least squares (PLS) regression models and its orthogonal variant (OPLS).When analyzing the wavelengths, at 280nm a greater differentiation between the models could be observed, *D. molliculum* presented two chromatographic peaks corresponding to tannic acid and 3-(3,4-dihydroxycinnamoyl) quinic acid (chlorogenic acid), not present in *D. adscendens*; while at 330nm, a higher concentration of vitexin was determined for *D. molliculum* extracts in comparison to *D. adscendens*. These differences may explain the greater antioxidant effect present in the extracts of *D*. molliculum species.

## Data Availability

The original contributions presented in the study are included in the article/**Supplementary Material**. Further inquiries can be directed to the corresponding author.
