# Supplementary figures and images for: Desmodium molliculum (Kunth) DC., an Andean medicinal plant: DNA barcoding and HPLC fingerprint for species discrimination and evaluation of its pharmacological potential
Source: Front Plant Sci. 2025 Jul 24;16:1612556. doi: 10.3389/fpls.2025.1612556 (PMC12328392; doi:10.3389/fpls.2025.1612556)

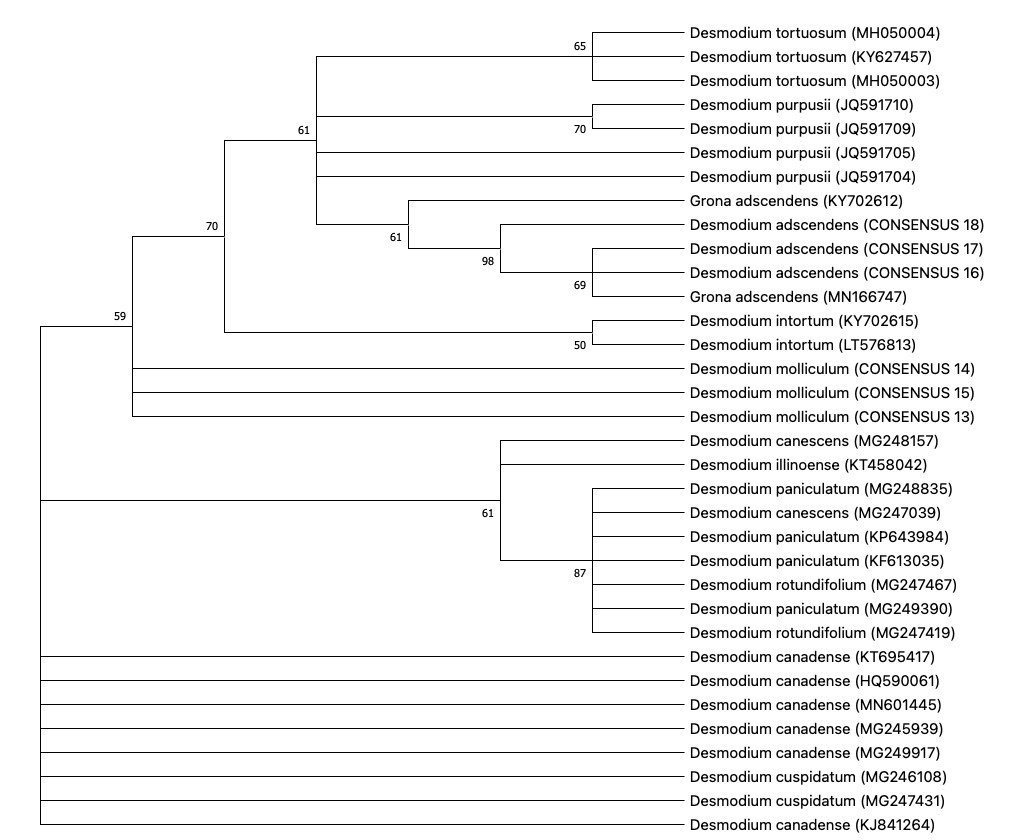

Supplement: Supplementary file 1 [file Image1.jpeg]

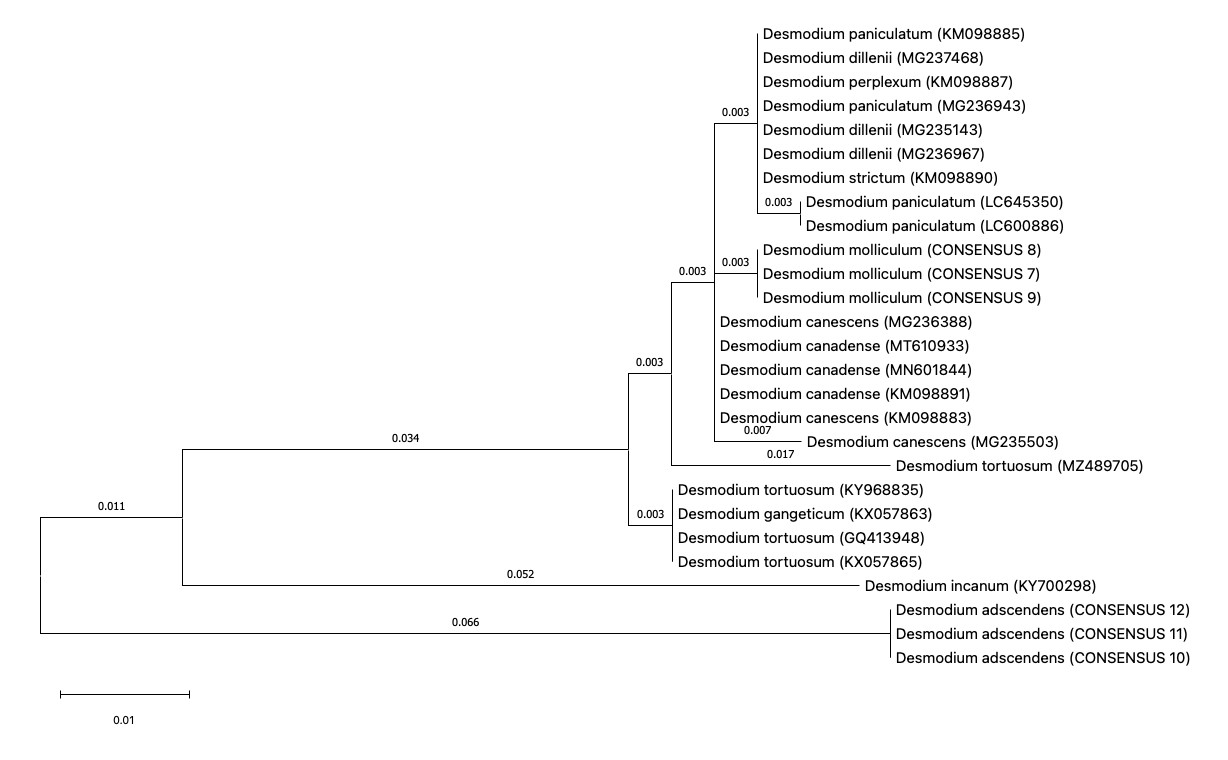

Supplement: Supplementary file 2 [file Image2.jpeg]

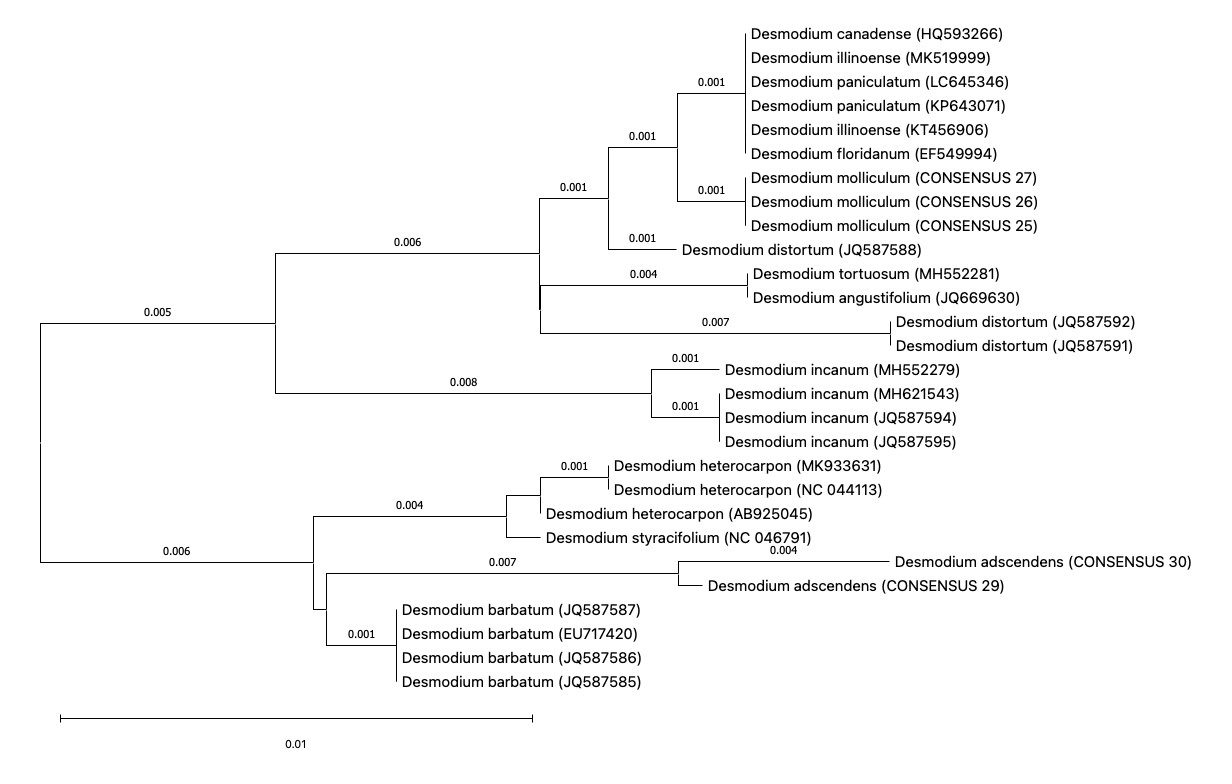

Supplement: Supplementary file 3 [file Image3.jpeg]

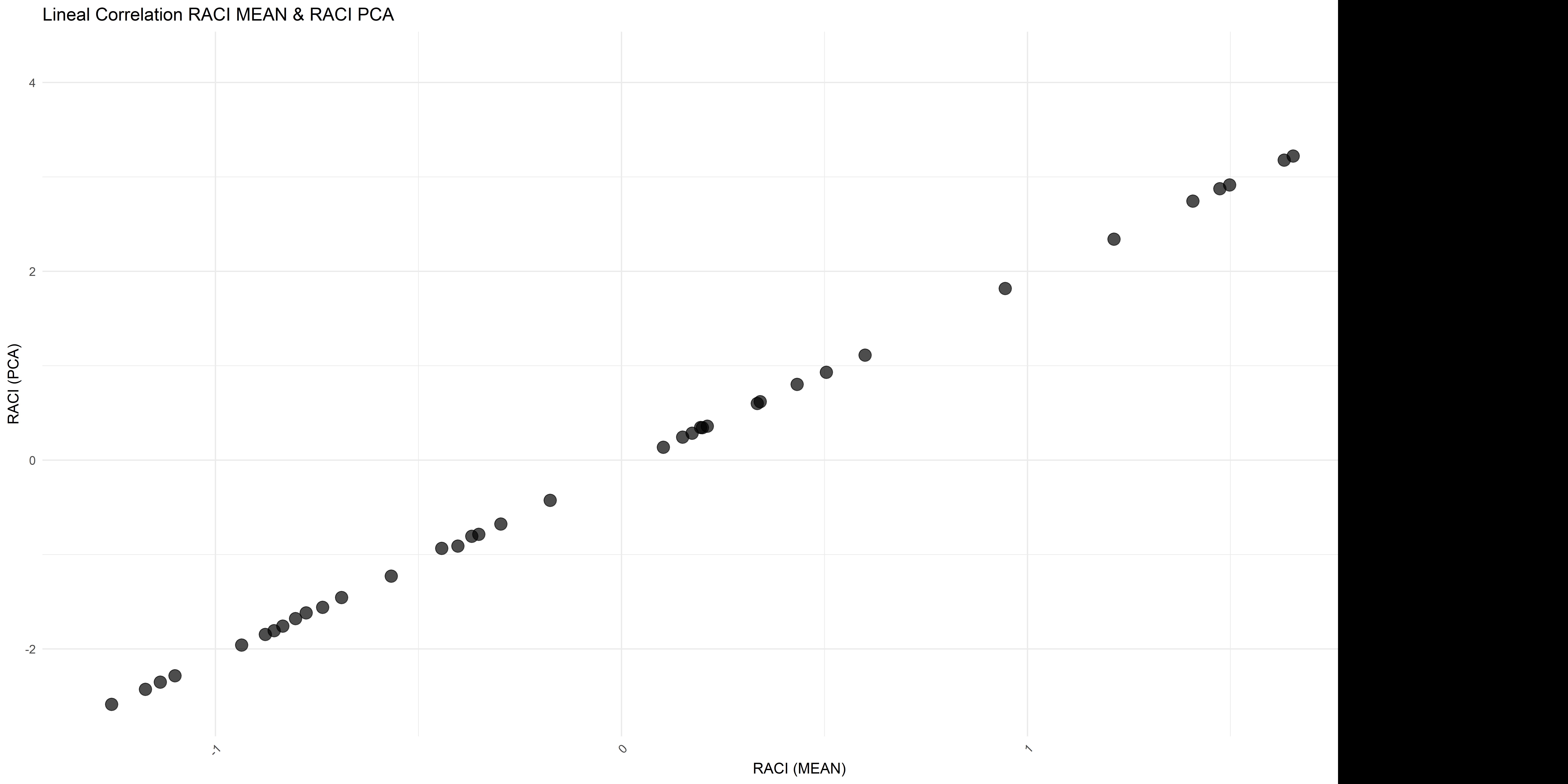

Supplement: Supplementary file 4 [file Image4.jpeg]
