# Supplementary material for: Desmodium molliculum (Kunth) DC., an Andean medicinal plant: DNA barcoding and HPLC fingerprint for species discrimination and evaluation of its pharmacological potential
Source: Front Plant Sci. 2025 Jul 24;16:1612556. doi: 10.3389/fpls.2025.1612556 (PMC12328392; doi:10.3389/fpls.2025.1612556)
Supplement: Supplementary file 5 [file Table1.docx]

| **PROJECT: "PHARMACOLOGICAL CHARACTERIZATION OF MEDICINAL PLANTS"** | | | | |
| --- | --- | --- | --- | --- |
| **COLLECTED SAMPLES** | | | | |
| ***Desmodium adscendens*** | | | **COORDINATES** | |
| **CODE** | **PLACE** | **ALTITUDE** | **X** | **Y** |
| **SAÁ 1** | EL ORO Atahualpa | **1405** | 646649 | 9602959 |
| **SAÁ 2** | EL ORO San Jacinto Calera chica | **1163** | 647868 | 9597617 |
| **SAÁ 3** | AZUAY Tendales | **1224** | 663847 | 9637006 |
| **SAÁ 4** | AZUAY San Antonio | **1277** | 670588 | 9635944 |
| **SAÁ 5** | AZUAY Tendales | **1230** | 663837 | 9637723 |
| **SAÁ 6** | AZUAY Tendales | **1305** | 663826 | 9637889 |
| **SAÁ 7** | AZUAY Tendales | **1240** | 663868 | 9637220 |
| **SAÁ 8** | EL ORO Santa Rosa | **1201** | 640828 | 9611332 |
| **SAÁ 9** | EL ORO Santa Rosa | **1335** | 640871 | 9610318 |
| **SAÁ 10** | EL ORO Santa Rosa | **1482** | 642344 | 9610254 |
| **SAÁ 11** | EL ORO Santa Rosa | **1102** | 641595 | 9611737 |
| **SAÁ 12** | EL ORO Santa Rosa | **1030** | 641580 | 9612733 |
| **SAÁ 13** | AZUAY Tamarindo | **1151** | 671951 | 9705832 |
| **SAÁ 14** | AZUAY Tamarindo | **1030** | 672699 | 9705475 |
| **SAÁ 15** | AZUAY Tamarindo | **1165** | 671856 | 9705861 |
| **SAÁ 16** | AZUAY Tamarindo | **1035** | 672699 | 9705475 |
| **SAÁ 17** | EL ORO Chilla | **1003** | 672699 | 9691220 |
| **SAÁ 18** | EL ORO Chilla | **1106** | 657986 | 9628077 |
| **SAÁ 19** | EL ORO Chilla | **1099** | 658040 | 9627697 |
| **SAÁ 20** | EL ORO Chilla | **1326** | 658588 | 9626457 |
| **SAÁ 21** | EL ORO Chilla | **1250** | 657896 | 9626314 |
| **SAÁ 22** | AZUAY Tendales | **1240** | 663868 | 9637220 |
| **SAÁ 23** | EL ORO Cerro Azul | **1472** | 642344 | 9610254 |

| **PROJECT: "PHARMACOLOGICAL CHARACTERIZATION OF MEDICINAL PLANTS"** | | | | |
| --- | --- | --- | --- | --- |
| **COLLECTED SAMPLES** | | | | |
| ***Desmodium molliculum*** | | | **COORDINATES** | |
| **CODE** | **PLACE** | **ALTITUDE** | **X** | **Y** |
| **CUZCO 1** | AZUAY Jadán-Jarata | **2632** | 735035 | 9679476 |
| **CUZCO 2** | AZUAY Jadán-Jarata | **2630** | 734823 | 9679380 |
| **CUZCO 3** | AZUAY Jadán-Jarata | **2618** | 734856 | 9679387 |
| **CUZCO 4** | AZUAY Jadán-Jarata | **2553** | 734877 | 9679059 |
| **CUZCO 5** | AZUAY Uzho | **2964** | 736974 | 9678201 |
| **CUZCO 6** | AZUAY Turi | **2725** | 720994 | 9675390 |
| **CUZCO 7** | AZUAY Turi | **2836** | 721608 | 9673497 |
| **CUZCO 8** | AZUAY Turi | **2872** | 721890 | 9670927 |
| **CUZCO 9** | AZUAY El Valle | **2651** | 728146 | 9674594 |
| **CUZCO 10** | AZUAY San Bartolomé | **2914** | 738267 | 9667085 |
| **CUZCO 11** | AZUAY San Bartolomé | **2688** | 741055 | 9668236 |
| **CUZCO 12** | AZUAY Barabón | **2748** | 713196 | 9677938 |
| **CUZCO 13** | AZUAY Barabón | **2803** | 710701 | 9676140 |
| **CUZCO 14** | AZUAY San Joaquín | **2725** | 714823 | 9680850 |
| **CUZCO 15** | AZUAY Sinincay | **2670** | 721217 | 9684151 |
| **CUZCO 16** | AZUAY Nero | **2858** | 715913 | 9674187 |
| **CUZCO 17** | AZUAY Nero | **2878** | 715964 | 9674354 |
| **CUZCO 18** | AZUAY Nero | **2856** | 709099 | 9675592 |
| **CUZCO 19** | AZUAY Urb. Vista Linda | **2555** | 719869 | 9677209 |
| **CUZCO 20** | AZUAY Sayausí | **2841** | 713133 | 9682868 |
| **CUZCO 21** | AZUAY Sayausí | **2745** | 713847 | 9682218 |
| **CUZCO 22** | AZUAY San José Mesaloma | **2843** | 714152 | 9680493 |
| **CUZCO 23** | AZUAY San José Mesaloma | **2978** | 713530 | 9680811 |
| **CUZCO 25** | AZUAY Barabón | **2749** | 713196 | 9677938 |
| **CUZCO 26** | AZUAY San José Mesaloma | **2978** | 713530 | 9680811 |
| **CUZCO 27** | AZUAY Jadán-Jarata | **2553** | 734877 | 9679059 |
