# Supplementary material for: Desmodium molliculum (Kunth) DC., an Andean medicinal plant: DNA barcoding and HPLC fingerprint for species discrimination and evaluation of its pharmacological potential
Source: Front Plant Sci. 2025 Jul 24;16:1612556. doi: 10.3389/fpls.2025.1612556 (PMC12328392; doi:10.3389/fpls.2025.1612556)
Supplement: Supplementary file 6 [file DataSheet1.docx]

**Figure S1:** Topology of the *rbcl* original tree.

**Figure S2:** Topology of the ITS2 original tree.

**Figure S3:** Topology of the *matK* original tree.

**Figure S4:** Correlation between RACI indexes (PCA vs mean)
